# Biotransformation of d-Xylose-Rich Rice Husk Hydrolysate by a Rice Paddy Soil Bacterium, *Priestia* sp. Strain JY310, to Low Molecular Weight Poly(3-hydroxybutyrate)

**DOI:** 10.3390/biom13010131

**Published:** 2023-01-09

**Authors:** Jae-Yeong Lee, Min-Hwan Kim, Jong-Sik Kim, Bo-Ram Yun, Do Young Kim, Chung-Wook Chung

**Affiliations:** 1 Department of Biological Sciences, Andong National University, Andong 36729, Republic of Korea; rnrtn5326@hanmail.net (J.-Y.L.); kmhkke2@naver.com (M.-H.K.); jsk@anu.ac.kr (J.-S.K.); 2 Korea Disease Control and Prevention Agency, Cheongju 28159, Republic of Korea; boram2183@korea.kr; 3 Microbiome Convergence Research Center, Korea Research Institute of Bioscience and Biotechnology(KRIBB), Daejeon 34141, Republic of Korea

**Keywords:** *Priestia* sp., thermochemical hydrolysis, rice husk hydrolysate, biotransformation, low molecular weight, poly(3-hydroxybutyrate), PHB

## Abstract

Poly(3-hydroxybutyrate) (PHB) is a versatile thermoplastic with superior biodegradability and biocompatibility that is intracellularly accumulated by numerous bacterial and archaeal species. *Priestia* sp. strain JY310 that was able to efficiently biotransform reducing sugars in d-xylose-rich rice husk hydrolysate (reducing sugar_RHH_) to PHB was isolated from the soil of a rice paddy. Reducing sugar_RHH_ including 12.5% d-glucose, 75.3% d-xylose, and 12.2% d-arabinose was simply prepared using thermochemical hydrolysis of 3% H_2_SO_4_-treated rice husk for 15 min at 121 °C. When cultured with 20 g/L reducing sugar_RHH_ under optimized culture conditions in a batch bioreactor, *Priestia* sp. strain JY310 could produce PHB homopolymer up to 50.4% of cell dry weight (6.2 g/L). The melting temperature, heat of fusion, and thermal decomposition temperature of PHB were determined to be 167.9 °C, 92.1 J/g, and 268.1 °C, respectively. The number average and weight average molecular weights of PHB with a broad polydispersity index value (4.73) were estimated to be approximately 16.2 and 76.8 kg/mol, respectively. The findings of the present study suggest that *Priestia* sp. strain JY310 can be exploited as a good candidate for the low-cost production of low molecular weight PHB with improved biodegradability and reduced brittleness from inexpensive agricultural waste hydrolysates.

## 1. Introduction

Petroleum-based plastics are among the most necessary materials in our daily life. They are closely related to a wide range of essential industries, such as aerospace, medical, automotive, and telecommunications [1]. As the use of single-use plastics has surged since the COVID-19 pandemic in 2019, global environmental problems caused by non-degradable plastics have become more serious [2]. It has been evaluated that between 4.8 and 12.7 million tonnes of plastic waste in landfills enter the ocean each year [3,4]. The projected plastic waste without improved waste management is expected to exceed 1.3 billion tonnes by 2040. In addition, as synthetic plastics are mainly produced from petroleum, which is a non-renewable resource, there are concerns about resource depletion and continuous use [3,5]. Therefore, there is a rapidly increasing demand for alternatives to petroleum-based plastics [6].

Diverse biodegradable biopolymers such as polylactide, poly(3-hydroxyalkanoates) (PHAs), and polypropiolactone have been developed as alternatives to petroleum-based synthetic plastics that are difficult to biologically degrade in natural environments [6,7,8,9]. The global production capacities of bioplastics are expected to increase from 2.42 million tonnes in 2021 to 7.59 million tonnes in 2026 (https://docs.european-bioplastics.org/publications/EUBP_Facts_and_figures.pdf, accessed on 23 October 2022). In particular, the percentage of the global production capacity of PHAs among biodegradable bioplastics is expected to increase from 1.8% in 2015 to 6.4% in 2026. Microbial poly(3-hydroxybutyrate) (PHB) is one of such versatile PHAs with physico-chemical properties similar to recalcitrant synthetic polymers, including polyethylene and polypropylene [10,11,12]. Moreover, due to its excellent biodegradability, biocompatibility, and low toxicity [13,14], PHB has drawn much attention as a promising material for biotechnological applications in food, agriculture, and environmental and biomedical industries [3,8]. However, compared to polypropylene, PHB has difficulties in commercialization due to its low recovery rate, low productivity, and the high production cost of the downstream process [15]. Therefore, for low-cost fermentative production of PHB by microorganisms, the main carbon source, which generally accounts for about 50% or more of the cost of PHB production [16], needs to be replaced with an inexpensive complex carbon source, such as agricultural byproducts, sewage sludge, and dairy waste [17,18,19]. Rice straw, sugar cane, and starch are examples of cheap carbon sources introduced for reducing the cost of PHB production [19,20,21]. In addition, like crops and forestry waste [22,23], alkali and ultrasound-pretreated rice straw have been reported as favorable complex carbon sources to reduce the cost of PHB production [24].

In 2017, the Food and Agriculture Organization of the United Nations estimated that the global production was approximately 769.6 million tonnes for rice and 150.0 million tonnes for rice husk (RH) (https://www.fao.org/3/I7658E/I7658E.pdf, accessed on 23 October 2022). As a cheap lignocellulosic biomass, RH is an agricultural waste of rice (*Oryza sativa*) that is generated during the milling process. It accounts for 20–25% of the total weight of rice [25]. Herein, we report the production of low molecular weight PHB by *Priestia* sp. strain JY310 isolated from the soil of a rice paddy using reducing sugars in d-xylose-rich RH hydrolysate (reducing sugar_RHH_) generated by the thermochemical hydrolysis of RH as a low-cost carbon source. Due to its improved biodegradability and reduced brittleness [26], low molecular weight PHB has outstanding potential in various industrial applications. The optimization of various parameters for the thermochemical hydrolysis of RH and the material properties of the produced PHB are also described.

## 2. Materials and Methods

### 2.1. Preparation of Reducing Sugar_RHH_

RH was purchased from a rice mill in Andong, Republic of Korea. After removing impurities from RH by washing with distilled water, the resulting RH was dried at 70 °C for 24 h in a drying oven, followed by grinding using a vacuum blender (CompLife, Incheon, Republic of Korea). The preparation of reducing sugar_RHH_ with different compositions from RH was conducted by acid hydrolysis under various reaction conditions as follows. Different amounts of RH (150, 200, and 250 g/L) were firstly treated with H_2_SO_4_ at concentrations of 1, 2, and 3% (*v*/*v*) and then autoclaved at 121 °C for 15, 30, 60, and 90 min, respectively. After thermochemical hydrolysis of RH, reaction mixtures were neutralized by adding 3 M NaOH, followed by centrifugation at 8000× *g* for 20 min at 4 °C. Recovered liquid solutions containing reducing sugar_RHH_ were used in aerobic fermentation experiments as carbon sources.

### 2.2. Isolation of PHA-Producing Bacteria

For the isolation of heterotrophic bacteria capable of efficiently fermenting reducing sugar_RHH_ prepared from H_2_SO_4_ (3%, *v*/*v*)-treated RH (250 g/L) by autoclaving at 121 °C for 90 min for their growth and PHA production, three different soil samples were collected by digging the soil surface at a depth of >8 cm immediately after harvesting rice from paddy fields in Andong, Republic of Korea. Approximately 15 g of respective soil sample was then thoroughly suspended in 40 mL of distilled water by vigorous stirring for 5 min at room temperature. Thereafter, the suspension was allowed to stand for 20 min without stirring to precipitate solid components of soil. Enrichment cultivation of reducing sugar_RHH_-fermenting bacteria in a soil sample was carried out by shaking at 200 rpm for 3 d at 30 °C after inoculating 10 mL of the soil supernatant into a 500 mL Erlenmeyer flask containing 100 mL of liquid mineral salts medium. Each liter of basal medium (pH 7.0) included 20 g reducing sugar_RHH_, 9.0 g Na_2_HPO_4_·12H_2_O, 1.5 g KH_2_PO_4_, 0.5 g NH_4_Cl, 0.2 g MgSO_4_·7H_2_O, and 1 mL of trace element solution consisting of 9.70 g FeCl_3_, 10.33 g CaCl_2_·2H_2_O, 0.22 g CoCl_2_·6H_2_O, 0.16 g CuSO_4_·5H_2_O, 0.12 g NiCl_2_·6H_2_O, and 0.11 g CrCl_2_·6H_2_O per liter of 0.1 N HCl. To reduce microbial diversity, each liquid culture procedure was consecutively repeated three times for 9 d. Briefly, 10 mL of enrichment broth culture, which was first prepared according to the aforementioned liquid culture procedure, was re-inoculated into the same fresh medium, followed by incubating under the same culture conditions. Thereafter, the liquid cultivation procedure was performed to enrich dominant reducing sugar_RHH_-fermenting bacterial species in the culture broth once again. Reducing sugar_RHH_-fermenting bacteria were selectively isolated as follows. A 100 μL aliquot of the culture broth was serially diluted up to 10^−5^ using a sterile liquid medium without reducing sugar_RHH_, after which a 50 μL aliquot of the diluted suspension was spread on a reducing sugar_RHH_-containing MSM agar plate and incubated at 30 °C for 3 d. Respective bacterial colonies showing different morphological characteristics formed on the solid medium were purely transferred to a new solid medium, after which isolates were incubated at 30 °C for 3 d. Of the isolated reducing sugar_RHH_-utilizing bacteria, strain JY310, which was identified as a superior PHB-producing candidate by pre-tests, was preferentially selected for further study. Quantitative analysis of PHB accumulated in bacterial isolates was performed by gas chromatography (GC).

### 2.3. Identification of a PHB-Producing Bacterial Isolate

Phylogenetic identification of strain JY310 was carried out using sequence analysis of its 16S rRNA gene. For this, genomic DNA of the isolate was extracted using a G-Spin Total DNA Extraction Kit (iNtRON Biotechnology, Inc., Seongnam, Republic of Korea) in accordance with the manufacturer’s protocol. The 16S rRNA gene of strain JY310 was amplified by polymerase chain reaction (PCR) with two universal primers of 8F (5′-AGAGTTTGATCMTG-GCTCAG-3′) and 1492R (5′-TACGGYTACCTTGTACGACTT-3′). With a 2X Thumb *Taq* PCR Pre-Mix (BioFACT Co., Ltd., Daejeon, Republic of Korea), PCR was carried out using a T100 thermal cycler (Bio-Rad Laboratories, Inc., Hercules, CA, USA) with the following cycling conditions: initial template denaturation at 94 °C for 2 min, followed by 30 cycles of 94 °C for 30 s, 55 °C for 30 s, and 72 °C for 1 min. The resulting PCR products were purely isolated using a NucleoSpin Gel and PCR Clean-up (Macherey-Nagel, Düren, Germany) and then sequenced with the aforementioned oligonucleotide primers. Using MEGA 11 software (https://www.megasoftware.net, accessed on 28 October 2022), the nucleotide sequence of its 16S rRNA gene was compared with those of strains deposited in the National Center for Biotechnology Information (NCBI) database to find closely related species.

### 2.4. Effects of Culture Conditions on Bacterial Growth and PHB Production

To examine the effects of culture temperature on the growth of strain JY310 and its PHB production, the bacterial cultivation was performed using a 500 mL Erlenmeyer flask, which contained 100 mL of liquid mineral salts medium (pH 6.0), in a rotary shaker (200 rpm) for 60 h at 20, 25, 30, 35, and 40 °C, respectively. As a carbon source, 20 g/L of reducing sugar_RHH_ prepared by autoclaving 3% H_2_SO_4_-treated RH for 15 min at 121 °C was added to the culture medium. However, the effects of medium pH on the growth and PHB biosynthesis of strain JY310 were investigated by culturing it at 30 °C with pH ranging from 5.0 to 9.0 under the aforementioned culture conditions with minor modifications. Flask cultures of strain JY310 were also conducted at 50, 100, 150, 200, and 250 rpm, respectively, to evaluate the effects of shaking speed on its growth and PHB production. In this case, the culture temperature of strain JY310, medium pH, and amount of reducing sugar_RHH_ were adjusted to 30 °C, 6.0, and 20 g/L, respectively. The effects of reducing sugar_RHH_ concentration in culture broth on the growth and PHB biosynthesis of the microorganism was assessed by growing it with the substrate at concentrations of 5, 10, 15, 20, 25, and 30 g/L, respectively, in a rotary shaker (200 rpm) for 60 h at 30 °C and pH 6.0. The effects of carbon to nitrogen (C/N) ratio in the range of 10–60 in culture medium on the growth and PHB production of strain JY310 were also analyzed. In this case, the microorganism, which was fed with 20 g/L of reducing sugar_RHH_, was cultivated for 60 h under the following culture conditions: temperature of 30 °C, pH 6.0, and shaking at 200 rpm. After the completion of cultivation, the cells were harvested by centrifugation at 13,000× *g* for 10 min at 4 °C, followed by lyophilization.

### 2.5. Batch Fermentation of Strain JY310

Using liquid mineral salts medium (C/N ratio: 40) supplemented with 20 g/L of reducing sugar_RHH_, which was prepared by thermochemical hydrolysis of 3% H_2_SO_4_–treated RH at 121 °C for 15 min, an optimized batch fermentation experiment was performed in a 3 L jar fermentor (Biofors Global Inc., Bucheon, Republic of Korea) with a working volume of 2 L. Fermentor culture of strain JY310 was initiated by inoculating with a 5% (*v*/*v*) inoculum of its overnight culture grown in nutrient broth (BD Difco, Franklin Lakes, NJ, USA), followed by incubating for 66 h. The pH, temperature, agitation speed, and aeration rate were automatically controlled at 6.0, 30 °C, 200 rpm, and 1.0 vvm, respectively. During batch fermentation, the culture broth samples of strain JY310 were taken at every 12 h to estimate its growth at 600 nm and ability to produce PHB, after which they were centrifuged at 13,000× *g* for 10 min at 4 °C. The resulting cell pellets were lyophilized, and the recovered culture supernatants were stored at 4 °C for further analysis of residual carbon and nitrogen sources. The batch fermentation was finished at approximately 2 h after the bacterial growth reached the stationary phase. The culture broth was then centrifuged at 13,000× *g* for 10 min at 4 °C.

### 2.6. Isolation and Purification of PHA

The PHA produced by strain JY310 was isolated from the lyophilized cells with hot chloroform employing a Soxhlet extractor. To prepare a fine product, the extracted crude PHA was precipitated by dropping into vigorously stirred cold methanol in a fume hood. This precipitation process was repeated at least three times. The resulting purified PHA was left in the fume hood for 3 d to evaporate remaining organic solvents. It was then used for further analysis.

### 2.7. Analytical Methods

The composition of monosaccharides in RH hydrolysates and residual amounts of monosaccharides in the culture supernatant were quantitatively analyzed by high-performance liquid chromatography (HPLC) with d-glucose, d-xylose, and d-arabinose as standards [27]. HPLC analysis was performed employing a Waters Alliance 2690 HPLC system (Waters Corp., Milford, MA, USA) equipped with a Sugar-Pak I column (10 μm, 6.5 mm × 300 mm, Waters Corp.) and a refractive index (RI) detector. The column temperature and sample injection volume used were 90 °C and 20 μL, respectively. A mobile phase consisting of 0.01 M Ca-EDTA was used at a flow rate of 0.5 mL/min. Quantitative determination of growth inhibitory substances present in RH hydrolysate was performed using furfural (Merck Millipore, Darmstadt, Germany) and 5-hydroxymethylfurfural (5-HMF) (TCI Co., Ltd., Tokyo, Japan) as standards by HPLC analysis with a Shim-pack VP-ODS column (5 μm, 4.6 × 250 mm, Shimadzu Corp., Kyoto, Japan). A mobile phase contained water and acetonitrile at a ratio of 8:2. The column temperature, sample injection volume, and flow rate were 40 °C, 10 μL, and 1 mL/min, respectively.

Colorimetric determination of residual NH_4_Cl in the culture supernatant was carried out according to the Nessler method [28]. For this, a standard calibration curve for NH_4_Cl was constructed by plotting the mean absorbance at 490 nm against NH_4_Cl concentration. It was then used for ammonium quantification. The standard reaction mixture (5.0 mL) contained 2.0 mL of Nessler reagent (Kanto Chemical Co., Inc., Tokyo, Japan), 2.95 mL of distilled water, and 0.05 mL of the culture supernatant.

Quantitative analysis of PHAs in lyophilized cells was conducted using GC with a GC-2010 Plus gas chromatograph (Shimadzu Corp., Kyoto, Japan) connected to an HP-1 capillary GC column (0.5 µm, 25 m × 0.2 mm, Agilent Technologies, Inc., Wilmington, DE, USA) and a flame ionization detector. For this, 20 mg of lyophilized cells was added to a PYREX screw cap culture tube with a PTFE lined phenolic cap (13 mm × 100 mm) containing a mixture of 1.0 mL chloroform, 0.85 mL methanol, 0.15 mL H_2_SO_4_, and 4 mg benzoic acid as an internal standard. The reaction mixture was then heated at 100 °C for 3 h. After methanolysis of cells, 1.0 mL of distilled water was added to the cold reaction mixture and then mixed vigorously to isolate the chloroform layer, including methyl esters of 3-hydroxyalkanoic and benzoic acids. The organic phase was carefully taken and analyzed by GC as described above. The oven temperature was initially kept at 80 °C for 4 min, after which it was increased at a rate of 10 °C/min to 230 °C. Identification of PHA monomeric units in methanolyzed samples was conducted using gas chromatography–mass spectrometry (GC-MS) analysis employing an Agilent 5977A Series GC/MSD system (Agilent Technologies, Inc., Santa Clara, CA, USA) equipped with an Agilent J&W DB-5MS GC column (0.25 μm, 30 m × 0.25 mm, Agilent Technologies, Inc.) under the aforementioned conditions. Structural identification of a PHA biosynthesized by strain JY310 was also performed by 600 MHz ^1^H nuclear magnetic resonance (NMR) spectroscopy analysis with a Bruker AVANCE III 600 NMR spectrometer (Bruker Corp., Billerica, MA, USA). A PHB obtained from Sigma-Aldrich (St. Louis, MO, USA) was used as a standard.

Molecular weight and its distribution of PHA were determined using size exclusion chromatography (SEC) with a Waters Alliance e2695 SEC system (Waters Corp.) connected with an RI detector. Approximately 5 mg of purified PHA dissolved in 1 mL tetrahydrofuran was filtered with a 0.45 μm PTFE syringe filter, after which 50 μL of the sample was injected into Waters Styragel columns (HR3, HR4, and HR5E) with oven temperature set at 35 °C using polystyrene standards (1060~3,580,000 Da) for calibration. Elution of PHA was performed using chloroform at a flow rate of 1 mL/min. Thermal behavior of PHA was analyzed using differential scanning calorimetry (DSC) with a DSC 200 PC Phox (Netzsch-Gerätebau GmbH, Selb, Germany). The temperature was scanned from −50 to 200 °C at a heating rate of 5 °C/min. Thermogravimetry/differential thermal analysis (TG/DTA) of PHA to determine its thermal stability was accomplished using a TG-DTA 8122 thermal analyzer (Rigaku Corp., Tokyo, Japan) at a heating rate of 10 °C/min under nitrogen atmosphere. The temperature used for TG/DTA ranged from 20 to 900 °C.

## 3. Results and Discussion

### 3.1. Phylogenetic Identification of a PHB-Accumulating Bacterial Isolate

**Figure 1:**
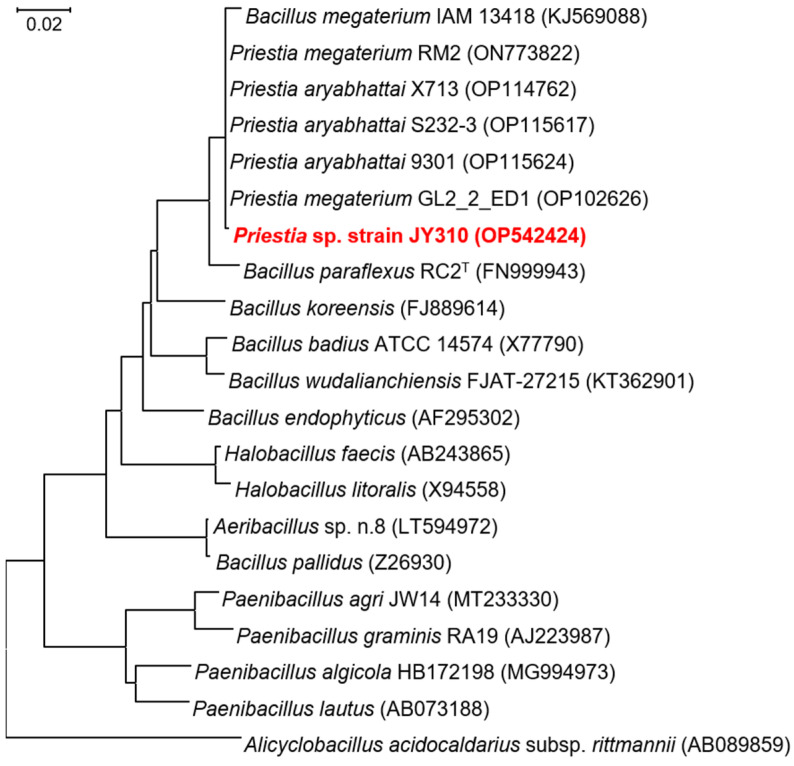
Neighbor-joining tree based on 16S rRNA gene sequences exhibiting the phylogenetic position of strain JY310 among its closely related strains. The nucleotide sequences used for phylogenetic analysis were retrieved from the GenBank database. Bar, 0.02 substitutions per nucleotide position.

A Gram-positive, aerobic, motile, and rod-shaped bacterium, strain JY310, which was efficiently able to biotransform reducing sugar_RHH_ (20 g/L) to PHB, was selectively isolated from a rice paddy soil using enrichment. The phylogenetic analysis of the strain JY310 revealed that its 16S rRNA gene sequence (GenBank accession number: OP542424) shared a sequence similarity of 99.85% with 16S rRNA gene sequences of some prokaryotes belonging to the genus *Priestia* (Figure 1). Moreover, a phylogenetic tree displaying the relationship between strain JY310 and its closely related relatives, exhibited in Figure 1, indicated that strain JY310 was differentiated from the strains of recognized *Priestia* species. Based on these results, strain JY310 was identified as a new species belonging to the genus *Priestia* and deposited in the Korean Collection for Type Cultures with a name of *Priestia* sp. strain JY310 KCTC 43440.

### 3.2. Preparation of Fermentable Reducing Sugar_RHH_ for PHB Production

**Figure 2:**
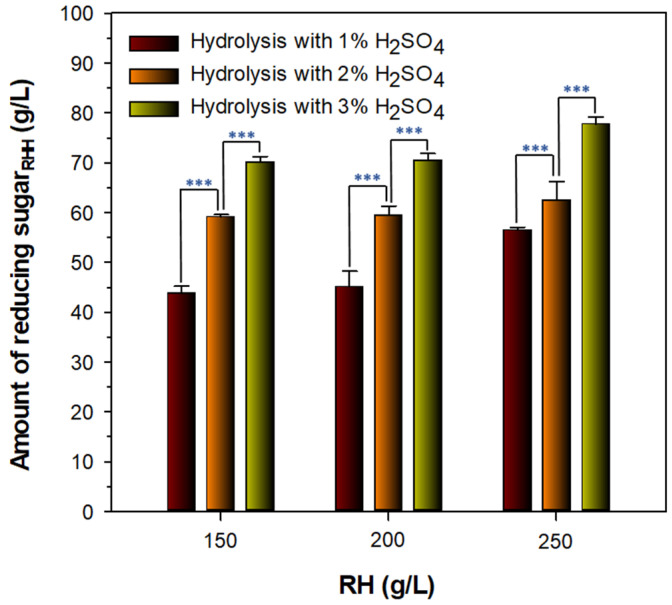
Thermochemical hydrolysis of different RH samples treated with H_2_SO_4_ at concentrations of 1, 2, and 3%, respectively. The values are mean ± SD of triplicate tests. *p*-Values were calculated using two-tailed Student’s *t*-test: *** *p* < 0.001.

For the efficient preparation of reducing sugar_RHH_ from RH, its thermochemical hydrolysis was performed by autoclaving for 90 min at 121 °C in the presence of 1, 2, and 3% H_2_SO_4_, respectively. As a result, it appeared that the acid hydrolysis of RH gradually increased when the concentration of H_2_SO_4_ in the reaction mixture was increased from 1% to 3%, regardless of the amount of RH evaluated (Figure 2).

Moreover, the preparation of reducing sugar_RHH_ from 3% H_2_SO_4_–treated RH could be maximally achieved when 250 g/L RH was subjected to thermochemical treatment that resulted in the production of 77 g/L reducing sugar_RHH_. Based on the above results, 250 g/L RH and 3% H_2_SO_4_ were preferentially selected as parameters for the optimal preparation of reducing sugar_RHH_ from the feedstock.

**Figure 3:**
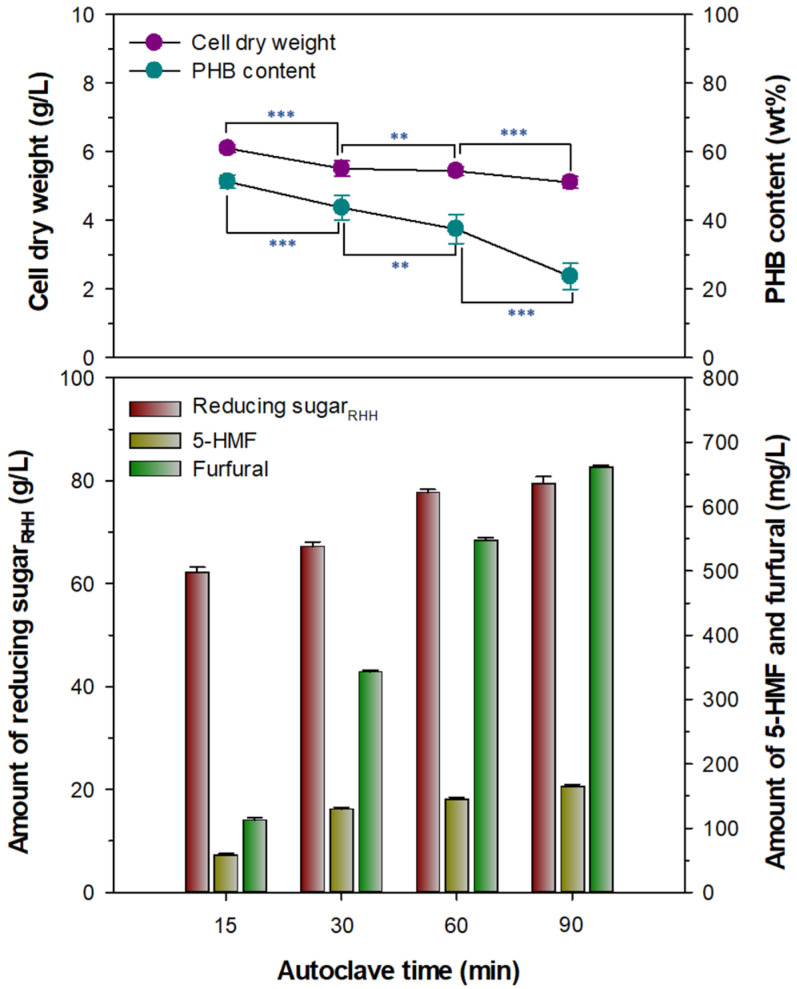
Effect of reducing sugar_RHH_ samples with different compositions, which were prepared by thermochemical hydrolysis of 3% H_2_SO_4_–treated RH in an autoclave time-dependent manner, on the growth and PHB production of *Priestia* sp. strain JY310. The values are mean ± SD of triplicate tests. *p*-Values were calculated using two-tailed Student’s *t*-test: ** *p* < 0.01 and *** *p* < 0.001.

It has been demonstrated that thermochemical treatments of lignocellulose at high temperatures in the presence of an acid catalyst generally accompany the formation of furfural and 5-HMF as undesired byproducts derived from the dehydration of hexose and pentose sugars, respectively [29]. Particularly, in the microbiological context, the furan molecules often downregulate the growth of diverse PHA-producing bacteria [30,31], although some natural and engineered PHA producers are not affected by these potent growth-inhibitory compounds [32,33,34]. Therefore, for the efficient biotransformation of lignocellulose hydrolysate into PHA, the formation of furfural and 5-HMF should be minimized during an acidic thermochemical process. Figure 3 clearly shows that during thermochemical hydrolysis of 250 g/L RH in the presence of 3% H_2_SO_4_, the generation of furfural and 5-HMF together with reducing sugar_RHH_ was greatly increased in an autoclave time-dependent manner. Specifically, after the autoclaving of 3% H_2_SO_4_–treated RH for 15, 30, 60, or 90 min, the amount of furfural formed in the reaction mixture was measured to be approximately 58, 129, 145, or 164 mg/L, respectively. The quantity of 5-HMF formed under the aforementioned reaction conditions was also found to be considerably increased from 112 to 660 mg/L in an autoclave time-dependent manner. When cultured with reducing sugar_RHH_ prepared by autoclaving for 15 min from 3% H_2_SO_4_-treated RH (250 g/L), *Priestia* sp. strain JY310 exhibited good growth during the culture period, with cell dry weight (CDW) and PHB content measured to be 6.1 g/L and 51.3 wt%, respectively (Figure 3).

However, the growth and PHB production of *Priestia* sp. strain JY310 were negatively affected when cultivated on reducing sugar_RHH_ prepared by autoclaving for 30, 60, or 90 min from 3% H_2_SO_4_-treated RH (250 g/L). These results might be closely related to the concentrations of furfural and 5-HMF in the culture medium. The aromatic organic compounds are known to inhibit the growth of various PHA producers in a dose-dependent manner [30,31]. Actually, the growth of *Priestia* sp. strain JY310 and its biotransformation efficiency of reducing sugar_RHH_ into PHB were observed to be gradually downregulated together with increases in furfural and 5-HMF concentrations in the culture broth (Figure 3). Based on these results, the adequate autoclave time of 3% H_2_SO_4_-treated RH (250 g/L) for the preparation of reducing sugar_RHH_ suitable for its growth and PHB biosynthesis was determined to be 15 min.

### 3.3. Optimization of Culture Conditions for Bacterial Growth and PHB Production

**Figure 4:**
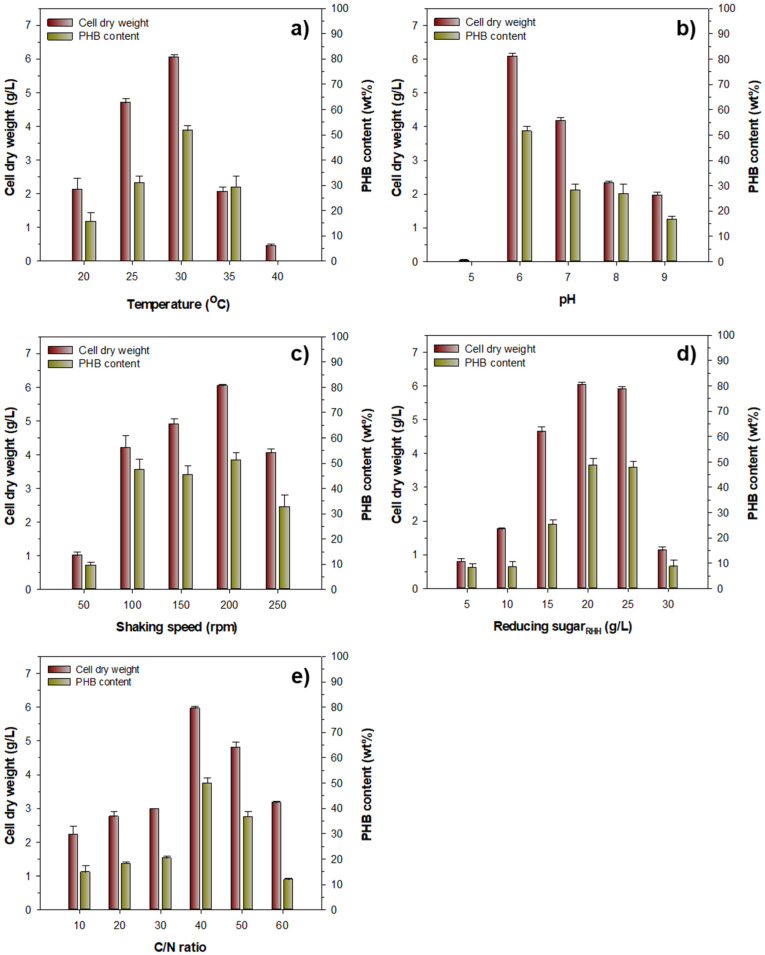
Effect of various culture conditions on the growth and PHB production of *Priestia* sp. strain JY310. The flask cultures of strain JY310 were performed for 60 h, as described in the Materials and Methods section. The optimal culture temperature (**a**) of the organism was examined at 20, 25, 30, 35, and 40 °C, respectively, and its optimal medium pH was determined in a pH range from 5.0 to 9.0 (**b**). The shaking speed (**c**) to optimize the growth and PHB production of strain JY310 was investigated at 20, 100, 150, 200, and 250 rpm, respectively. The optimal concentration of reducing sugar_RHH_ (**d**) in the culture medium to support its growth and PHB production was evaluated in a concentration range from 5 to 30 g/L. The optimal C/N ratio (**e**) was assessed in the range between 10 and 60. The values are mean ± SD of triplicate tests.

Similar to d-xylose-rich rice straw hydrolysate [35], reducing sugar_RHH_ containing 12.5% d-glucose, 75.3% d-xylose, and 12.2% d-arabinose in this study was employed as a cheap carbon source for the biosynthesis of PHB by *Priestia* sp. strain JY310. The optimization of PHB production by the microorganism was performed by determining various cultivation parameters (Figure 4). Of the tested culture temperatures, strain JY310 showed the maximum growth and PHB biosynthesis when it was cultured at 30 °C with 20 g/L reducing sugar_RHH_ prepared by the thermochemical hydrolysis of 3% H_2_SO_4_-treated RH for 15 min at 121 °C (Figure 4a). In this case, the CDW and PHB content were measured to be approximately 6.1 g/L and 51.7 wt%, respectively. However, after the cultivation of 60 h, it was observed that the bacterial growth and PHB production at temperatures (35 and 40 °C) above the optimal culture temperature were noticeably downregulated. It should also be noted that the optimal medium pH for the growth and PHB biosynthesis of *Priestia* sp. strain JY310 was found to be 6.0 (Figure 4b). Conversely, its growth was observed to be very slow at pH 5.0. It was also gradually downregulated when the microorganism was cultivated at pH values above the optimal pH value. It seems likely that *Priestia* sp. strain JY310 displayed optimal growth and PHB accumulation when it was aerobically grown on 20 g/L reducing sugar_RHH_ with a shaking speed of 200 rpm at 30 °C and pH 6.0 for 60 h (Figure 4c). In this case, the CDW and PHB content were determined to be approximately 6.0 g/L and 51.3 wt%, respectively. However, an increase in shaking speed from 200 to 250 rpm resulted in an approximately 33.2% decrease in cell growth together with a 36.5 wt.% reduction in PHB content in the cells. Meanwhile, at a concentration of 20 g/L, reducing sugar_RHH_ appeared to optimally support the growth and PHB biosynthesis of *Priestia* sp. strain JY310, although a similar result was also observed when 25 g/L reducing sugar_RHH_ was used (Figure 4d). In addition, it was found that a carbon-to-nitrogen (C/N) ratio of 40 most effectively supported both cell growth and PHB biosynthesis (Figure 4e). Based on the above results, the cultivation parameters for the optimal growth and PHB production of *Priestia* sp. strain JY310 were established as follows: culture temperature of 30 °C, medium pH of 6.0, shaking speed of 200 rpm, reducing sugar_RHH_ concentration of 20 g/L, and C/N ratio of 40.

### 3.4. Bacterial Production of PHB by Batch Fermentation under Optimized Culture Conditions

Recently, different studies on the cost-effective production of PHB by some bacterial species from various lignocellulose hydrolysates in shake flasks, batch bioreactors, or fed-batch bioreactors have been frequently reported (Table 1).

**Figure 5:**
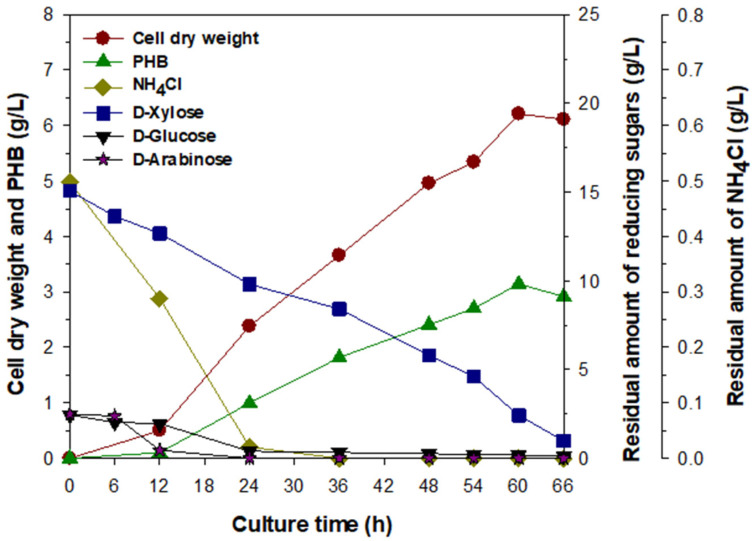
Fermentation kinetics of PHB production by *Priestia* sp. strain JY310 cultured with 20 g/L reducing sugar_RHH_.

In previous studies, lignocellulose hydrolysates for the bacterial production of PHB were generally prepared using the following methods: biological hydrolysis [43], thermochemical hydrolysis [21,33,38,44], thermochemical and enzymatic hydrolysis [34,35,39,40,41,42], the AFEX process and enzymatic hydrolysis [36], or thermomechanical pulping and enzymatic hydrolysis [38]. Accordingly, in this study, reducing sugar_RHH_ (20 g/L) simply prepared by the thermochemical hydrolysis of 3% H_2_SO_4_-treated RH was used as a cheap carbon source for the substantial production of PHB by *Priestia* sp. strain JY310 under optimized culture conditions (Figure 5). However, the production of PHB by *Burkholderia cepacia* USM [35] and *Cuprividus necator* [45] was performed with RH hydrolysates prepared by the enzymatic hydrolysis of alkali- and steam flash-explosion-treated RH, respectively. In particular, it has also been described that the difference in the preparation method of RH hydrolysates results in the formation of a mixture showing different sugar compositions [35,45].

During the batch fermentation process, the production of PHB in the cells was first detected in a small quantity (<0.1 g/L) after 12 h of cultivation, as shown in the production of PHB by *Paraburkholderia sacchari* (synonym *Burkholderia sacchari* [46]) IPT 101 with hardwood hydrolysate in a fed-batch bioreactor [38]. However, its growth and PHB production were markedly increased during the logarithmic phase, accompanied by a continuous consumption of reducing sugar_RHH_ (12.5% d-glucose, 75.3% d-xylose, and 12.2% d-arabinose). Especially, the complete consumption of d-glucose by *Priestia* sp. strain JY310 was observed before a cultivation period of 12 h, while most d-xylose and d-arabinose in the medium were continuously uptaken by the organism during the batch fermentation, as determined by HPLC analysis. In this case, the maximum CDW and PHB accumulation of *Priestia* sp. strain JY310 analyzed after a cultivation period of 60 h were estimated to be 6.2 and 3.1 g/L, respectively. These results were very comparable to those of the PHB production by some other bacteria from different lignocellulose hydrolysates prepared by thermochemical hydrolysis (Table 1). Previously, it has been reported that *B. cepacia* IPT 048 and *B. sacchari* IPT 101 can grow by 4.4 g/L of CDW together with a PHB accumulation of 2.3 and 2.7 g/L, respectively, when cultured with sugarcane bagasse hydrolysate in a batch bioreactor [21]. In addition, the CDW and PHB accumulation of *Halomonas halophila* CCM 3662 grown with spent coffee grounds hydrolysate were determined to be 3.5 and 2.1 g/L, respectively [44]. Furthermore, the amount (3.1 g/L) of PHB produced by *Priestia* sp. strain JY310 from reducing sugar_RHH_ was approximately 1.9-fold higher than that (1.6 g/L) of PHB biosynthesized by *Bacillus firmus* NII 0830 from rice straw hydrolysate [33]. The above descriptions suggest that *Priestia* sp. strain JY310 is a potential candidate capable of efficiently producing PHB from reducing sugar_RHH_, which can be simply prepared by autoclaving 3% H_2_SO_4_-treated RH for 15 min at 121 °C. Meanwhile, it has been demonstrated that lignocellulose hydrolysates prepared by both thermochemical and enzymatic hydrolysis processes support the bacterial growth and PHB biosynthesis better than those made by thermochemical hydrolysis processes (Table 1). For example, the amount (3.9 g/L) of PHB produced by *B. cepacia* USM [35] from rice husk hydrolysate in a batch bioreactor was approximately 1.2-fold higher than that (3.2 g/L) of PHB produced by *Priestia* sp. strain JY310 from reducing sugar_RHH_. Moreover, amounts of PHB biosynthesized by *Ralsonia eutropha* NCIMB 11599 [39] from wheat bran hydrolysate and *R. eutropha* ATCC 17699 [40] from rice paddy straw hydrolysate were assessed to be 15.3 and 9.8 g/L, respectively. Nevertheless, it is considered that compared to the known thermochemical hydrolysis processes of lignocellulosic biomass (Table 1), the thermochemical and enzymatic hydrolysis processes to make lignocellulose hydrolysates have some disadvantages, such as process complexity and enzyme costs.

### 3.5. Characterization of PHB Biosynthesized by Priestia sp. Strain JY310

**Figure 6:**
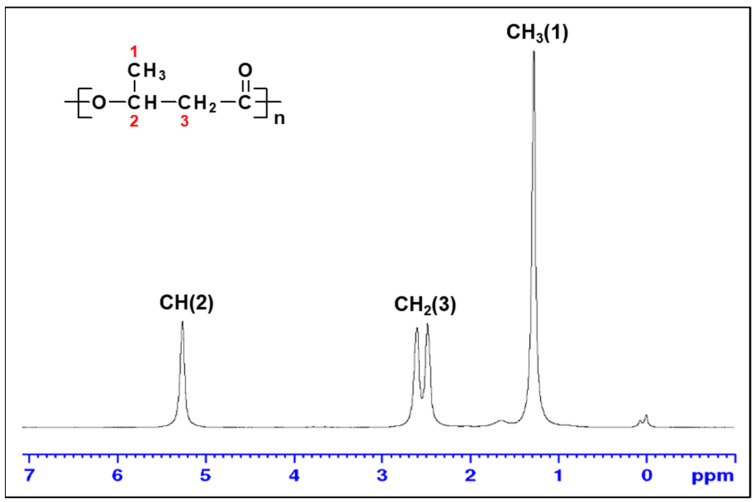
^1^H NMR spectrum (600 MHz) of a PHA biosynthesized by *Priestia* sp. strain JY310.

The ^1^H NMR spectrum of a PHA sample biosynthesized by *Priestia* sp. strain JY310 from reducing sugar_RHH_ is shown in Figure 6. It was found that the chemical shifts and patterns of peaks in the spectrum coincided well with those expected from a commercial PHB standard, indicating that the obtained PHA was a PHB homopolymer consisting of only 3-hydroxybutyrate repeating units.

**Figure 7:**
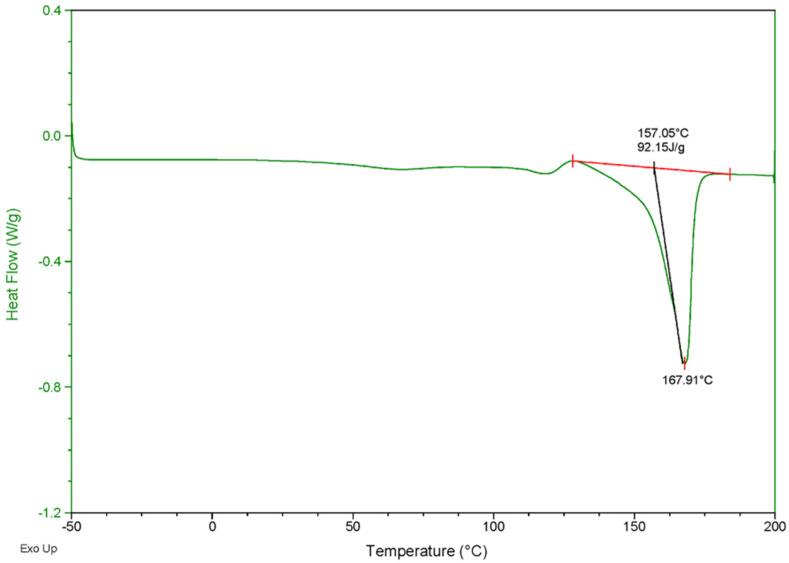
DSC thermogram of PHB biosynthesized by *Priestia* sp. strain JY310.

**Figure 8:**
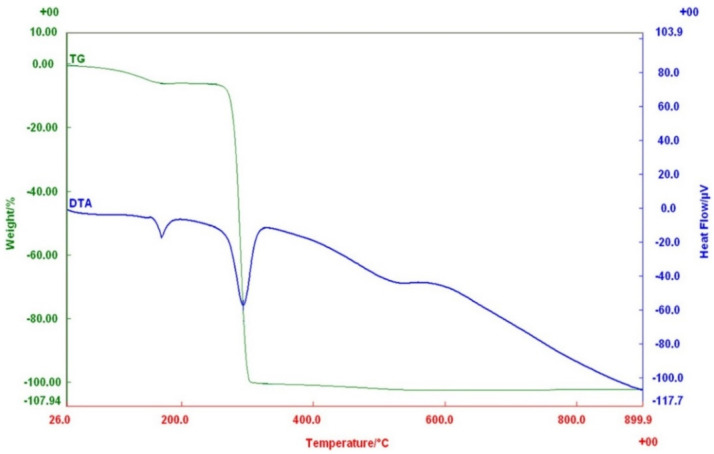
TG/DTA thermogram of PHB biosynthesized by *Priestia* sp. strain JY310 under nitrogen atmosphere.

**Figure 9:**
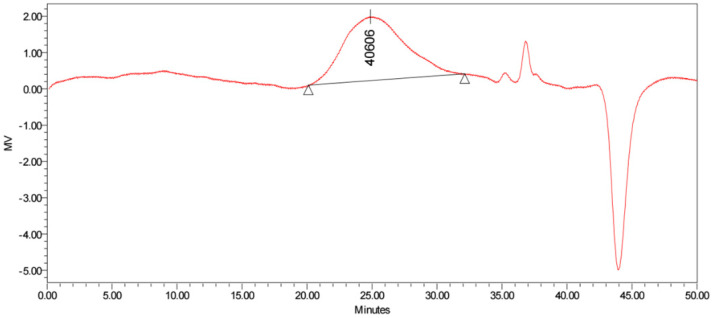
Size exclusion chromatogram showing molecular weight distribution of PHB produced by *Priestia* sp. strain JY310.

The result of thermal analysis clearly showed that the melting temperature (*T_m_*) and heat of fusion (Δ*H_m_*) of the PHB produced by *Priestia* sp. strain JY310 were 167.9 °C and 92.1 J/g, respectively, while its glass transition temperature (*T_g_*) was unclear (Figure 7). In addition, the TG/DTA thermogram clearly revealed that the decomposition temperature (*T_d_*) of PHB was 268.1 °C, and its thermal degradation was completed at 302.5 °C (Figure 8). Taken together, it should be noted that the thermal properties of PHB biosynthesized by *Priestia* sp. strain JY310 were noticeably different from those of standard PHB and other known PHB polymers (Table 2). For example, the *T_m_* (167.9 °C) and *T_d_* (268.1 °C) values of PHB produced by *Priestia* sp. strain JY310 were lower than those (*T_m_*: 176.0 °C and *T_d_*: 302.0 °C) of standard PHB [47,48]. Moreover, the *T_m_* and *T_d_* values of PHB produced by *C. necator* from RH hydrolysate have been reported to be 175.1 and 280.0 °C, respectively [49]. Furthermore, the *T_m_* of PHB accumulated in *Shewanella marisflavi* BBL25 [34] and *Loktanella* sp. SM43 [42] grown with barley straw and pine tree hydrolysates, respectively, was analyzed to be 176.7 °C. In particular, it was assessed that the *T_d_* (268.1 °C) of PHB biosynthesized by *Priestia* sp. strain JY310 was much lower than that (283.5 °C) of PHB produced by *R. eutropha* ATCC 17699 from rice paddy straw hydrolysate [40] and that (292.8 °C) of PHB extracted from the same organism grown with kenaf hydrolysate [41]. It is assumed that compared to the *T_m_* values (>171.5 °C) of other PHB polymers listed in Table 2, the lower *T_m_* (167.9 °C) of PHB produced by *Priestia* sp. strain JY310 might be due to its low molecular weight (Figure 9), as described previously [26].

It is of great interest to note that the number average molecular weight (*M_n_*), weight average molecular weight (*M_w_*), and peak molecular weight (*M_p_*) of PHB produced by *Priestia* sp. strain JY310 were 16.3, 76.8, and 40.6 kg/mol, respectively, by SEC (Figure 9, Table 2). The molecular weight and molecular weight distribution of the PHB were very comparable to those of standard PHB and other PHB polymers biosynthesized by different microorganisms from lignocellulose hydrolysates (Table 2). Especially, the *M_w_* (76.8 kg/mol) of PHB with a polydispersity index (PDI: *M_w_*/*M_n_*) value of 4.73, which was produced by *Priestia* sp. strain JY310, was significantly lower than that (1403 kg/mol) of PHB with an *M_w_*/*M_n_* value of 1.10 biosynthesized by *S. marisflavi* BBL25 [34] from barley straw hydrolysate. Additionally, the *M_w_* (810.0 kg/mol) of PHB with an *M_w_*/*M_n_* value of 1.58 produced by *Loktanella* sp. SM43 [42] from pine tree hydrolysate was much higher than that (76.8 kg/mol) of PHB accumulated in *Priestia* sp. strain JY310. It has been reported that the *M_w_* and *M_n_* of PHA polymers are commonly determined by the ratio of the PHA synthase gene (*phaC*) to 3-ketothiolase and acetyl-CoA reductase genes (*phaAB*) expression levels [49]. Therefore, it is considered that a big difference in the PDI values of the PHB polymers produced by *Priestia* sp. strain JY310 and other bacterial species [34,42,45] might be caused by differences in the expression levels of the aforementioned three genes among distinct PHB producers. Meanwhile, it has been reported that *Azotobacter vinelandii* is able to biosynthesize high and ultra-high *M_w_* PHB polymers with values between 2300 and 6600 kg/mol from sucrose [50]. Based on these results, it is suggested that the low *M_w_* PHB biosynthesized by *Priestia* sp. strain JY310 from reducing sugar_RHH_ is expected to be useful as an eco-friendly biomaterial with improved biodegradability and reduced brittleness for various industrial applications, as described by Hong et al. [26].

## 4. Conclusions

A rice paddy soil isolate, *Priestia* sp. strain JY310, efficiently biotransformed reducing sugar_RHH_ simply prepared by the thermochemical hydrolysis of RH to low *M_w_* PHB with a broad *M_w_*/*M_n_* value under the optimized culture conditions. Due to its ability to biosynthesize low *M_w_* PHB, the microorganism can be exploited as a suitable candidate for the production of diverse low *M_w_* thermoplastics with distinct biodegradability and brittleness that consist of either 3-hydroxybutyrate, 3-hydroxyvalerate, or in combination. Using various lignocellulose hydrolysates made by thermochemical hydrolysis together with thermochemical and enzymatic hydrolysis, batch and fed-batch fermentation experiments of *Priestia* sp. strain JY310 for the low-cost production of low *M_w_* PHB polymers are in progress.

## Figures and Tables

**Table 1:** Production of PHB by *Priestia* sp. strain JY310 and other bacterial species from lignocellulose hydrolysates.

Strain	Carbon Source	Preparation Method	CDW (g/L)	PHB Content (wt%)	PHB (g/L)	Scale	Reference
*Priestia* sp. strain JY310	Rice husk hydrolysate	Thermochemical hydrolysis	6.2	50.4	3.1	Batch bioreactor	This study
*Burkholderia cepacia * USM	Rice husk hydrolysate	Thermochemical and enzymatic hydrolysis	7.8	50.0	3.9	Batch Bioreactor	[35]
*Burkholderia cepacia* IPT 048	Sugarcane bagasse hydrolysate	Thermochemical hydrolysis	4.4	53.0	2.3	Batch bioreactor	[21]
*Burkholderia sacchari* IPT 101	Sugarcane bagasse hydrolysate	Thermochemical hydrolysis	4.4	62.0	2.7	Batch bioreactor	[21]
*Burkholderia sacchari* DSM 17165	Wheat straw hydrolysate	AFEX process and enzymatic hydrolysis	7.7	57.0	4.4	Fed-batch bioreactor	[36]
*Paraburkholderia sacchari* IPT 101	Softwood hemicellulose hydrolysates	Thermochemical hydrolysis	7.1	80.5	5.7	Shake Flask	[37]
*Paraburkholderia sacchari* IPT 101	Sugarcane hydrolysate	Thermochemical hydrolysis	40.3	55.0	22.0	Fed-batch bioreactor	[38]
*Paraburkholderia sacchari* IPT 101	Hardwood hydrolysate	Thermomechanical pulping and enzymatic hydrolysis	59.5	58.0	34.5	Fed-batch bioreactor	[38]
*Shewanella marisflavi * BBL25	Barley straw hydrolysate	Thermochemical and enzymatic hydrolysis	5.8	56.0	3.2	Shake Flask	[34]
*Ralstonia eutropha * NCIMB 11599	Wheat bran hydrolysate	Thermochemical and enzymatic hydrolysis	24.5	62.5	15.3	Shake Flask	[39]
*Ralstonia eutropha * ATCC 17699	Rice paddy straw hydrolysate	Thermochemical and enzymatic hydrolysis	15.5	63.7	9.8	Shake Flask	[40]
*Ralstonia eutropha * ATCC 17699	Kenaf hydrolysate	Thermochemical and enzymatic hydrolysis	17.9	81.0	10.1	Shake Flask	[41]
*Loktanella* sp. SM43	Pine tree hydrolysate	Thermochemical and enzymatic hydrolysis	4.68	78.0	3.6	Shake Flask	[42]
*Bacillus firmus * NII 0830	Rice straw hydrolysate	Thermochemical hydrolysis	1.9	89.0	1.6	Shake flask	[33]
*Bacillus megaterium* Ti3	Corn husk hydrolysate	Biological hydrolysis	1.7	59.0	1.0	Shake flask	[43]
*Halomonas halophila* CCM 3662	Spent coffee grounds hydrolysate	Thermochemical hydrolysis	3.5	61.9	2.1	Shake flask	[44]

**Table 2:** Thermal properties and molecular weights of some bacterial PHB polymers produced from lignocellulose hydrolysates.

Source	Carbon Substrate	*T_g_* (°C)	*T_m_* (°C)	*T_d_* (°C)	*M*_n_ (g/mol)	*M*_w_ (g/mol)	PDI (*M*_w_/*M*_n_)	Reference
*Priestia* sp. strain JY310	Rice husk hydrolysate	NI ^a^	167.9	268.1	16,200	76,800	4.73	This study
*Cupriavidus* necator	Rice husk hydrolysate	NI	175.1	280.0	124,000	135,000	1.09	[45]
*Shewanella marisflavi * BBL25	Barley straw hydrolysate	NI	176.7	ND ^b^	1,548,000	1,403,000	1.10	[34]
*Ralstonia eutropha * ATCC 17699	Rice paddy straw hydrolysate	10.0	172.2	283.5	ND	ND	ND	[40]
*Ralstonia eutropha * ATCC 17699	Kenaf hydrolysate	NI	171.6	292.8	ND	ND	ND	[41]
*Loktanella* sp. SM43	Pine tree hydrolysate	NI	176.7	ND	511,000	810,000	1.58	[42]
Standard PHB	-	NI	176.0	302.0	82,400	264,000	3.21	[46,47]

^a^ Not indicated; ^b^ not determined.

## Data Availability

Not applicable.
